# Thermochemical Ablation Therapy of VX2 Tumor Using a Permeable Oil-Packed Liquid Alkali Metal

**DOI:** 10.1371/journal.pone.0123196

**Published:** 2015-04-17

**Authors:** Ziyi Guo, Qiang Zhang, Xiaoguang Li, Zhengyu Jing

**Affiliations:** 1 Department of Radiology, Peking Union Medical College Hospital, Chinese Academy of Medical Sciences and Peking Union Medical College, Beijing, China; 2 Department of Radiology, Haikou People's Hospital, Xiangya Medical School Central South University, Haikou City, Hainan, China; RMIT University, AUSTRALIA

## Abstract

**Objective:**

Alkali metal appears to be a promising tool in thermochemical ablation, but, it requires additional data on safety is required. The objective of this study was to explore the effectiveness of permeable oil-packed liquid alkali metal in the thermochemical ablation of tumors.

**Methods:**

Permeable oil-packed sodium–potassium (NaK) was prepared using ultrasonic mixing of different ratios of metal to oil. The thermal effect of the mixture during ablation of muscle tissue ex vivo was evaluated using the Fluke Ti400 Thermal Imager. The thermochemical effect of the NaK-oil mixture on VX2 tumors was evaluated by performing perfusion CT scans both before and after treatment in 10 VX2 rabbit model tumors. VX2 tumors were harvested from two rabbits immediately after treatment to assess their viability using trypan blue and hematoxylin and eosin (H.E.) staining.

**Results:**

The injection of the NaK–oil mixture resulted in significantly higher heat in the ablation areas. The permeable oil controlled the rate of heat released during the NaK reaction with water in the living tissue. Perfusion computed tomography and its parameter map confirmed that the NaK–oil mixture had curative effects on VX2 tumors. Both trypan blue and H.E. staining showed partial necrosis of the VX2 tumors.

**Conclusions:**

The NaK–oil mixture may be used successfully to ablate tumor tissue *in vivo*. With reference to the controlled thermal and chemical lethal injury to tumors, using a liquid alkali in ablation is potentially an effective and safe method to treat malignant tumors.

## Introduction

Malignant tumors have become one of the most leading causes of death, and continue to affect more and more people throughout the world [1]. Thermal ablation techniques, such as radiofrequency (RF) [2], microwave [3], laser-induced thermal therapy [4], and high-intensity focused ultrasound [5], have emerged as less-invasive and more widely available alternatives; however, these techniques are expensive and not readily available.

The most commonly used agents in chemical ablation are 50% acetic acid and absolute ethanol [6]. Chemical ablation with ethanol or acetic acid is simple, inexpensive, and can be performed on an outpatient basis, but the toxicities in each case limit their applications [7]. Thermochemical methods are an alternative to thermal ablation. In an attempt to find a more localized, safer, and less-expensive alternative, a minimally invasive thermochemical therapy for tumors using alkali metals as powerful self-heating seeds was proposed by Rao and Liu [8–10]. The target tissue was treated using a direct injection of an extremely small amount of alkali metals. The reaction between the alkali metals and water in the target tissue produced a strong exothermic chemical reaction, and combustion, at the target site. The temperature in the target area induced tumor coagulation necrosis [10]. Hydroxyl ions were concurrently generated, and the alkaline environment enhanced the curative effects of targeted tumor ablation.

Because liquid sodium–potassium (NaK) alloy remains in a liquid state at room temperature [11], it can be delivered directly and noninvasively into the targeted tissue using a syringe; however, to ensure the safe use of a liquid alkali alloy in clinical practice, it must be adequately packed for a controlled release. The use of permeable oil is proposed to seal the NaK mixture, and control the release of the hydroxy radical and heat. Because of the reaction between sodium-potassium alloy-oil mixture and water in local biological tissues, this highly localized thermal chemical reaction is comparatively safe and beneficial for targeted tumor ablation, since heat and hydroxide are released only at the target with a high degree of controllability. In this report, permeable oil-sealed NaK was used to treat VX2 tumors *in vivo*. Both perfusion CT imaging and pathology assessments evaluated the curative effects of the NaK–oil mixture.

## Material and Methods

### Functionalized and permeable oil-sealed NaK

**Fig 1:**
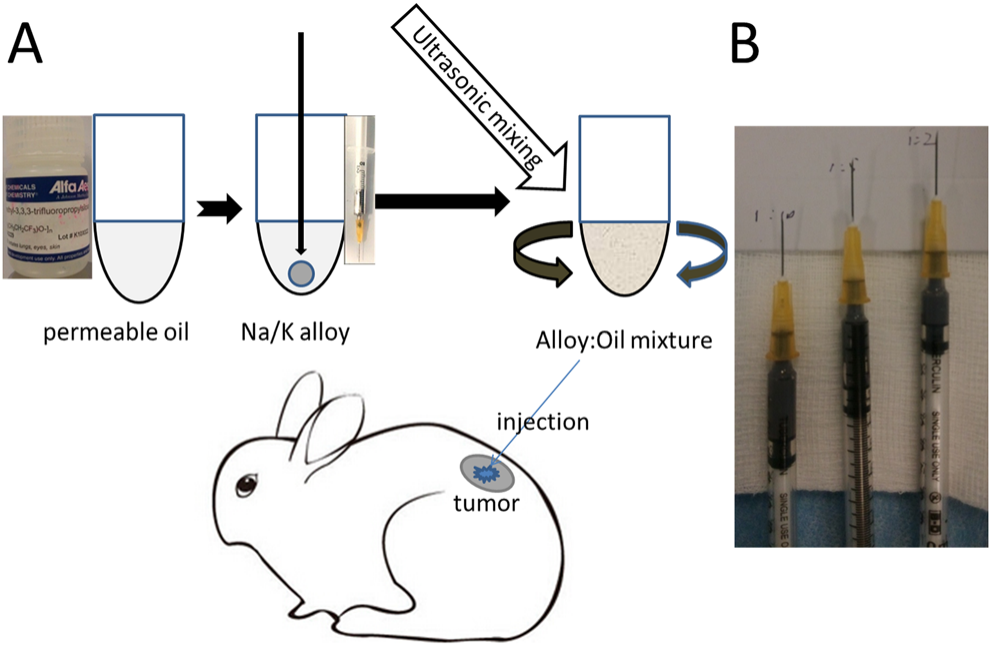
Flow chart for the preparation of the sodium-potassium (NaK) permeable-oil mixture. (A) first, accurately combine the permeable oil (FMS-121) and NaK in accordance with the volume ratio; second, inject NaK into the bottom of a 1.5-mL EP tube immersed in oil, and then carefully mix using ultrasonic probe vibration. (B) Prepare various ratios of the NaK–oil mixture encapsulated in syringes.

NaK is made by stirring Na and K at a 1:1 ratio under nitrogen at room temperature in a glass jar. Because NaK is a liquid at room temperature, it could be homogeneously mixed with polytrifluoropropylmethylsiloxane homopolymer (FMS-121, Alfa Aesar), which is water permeable [12], using the SKL150-IIN ultrasonic processor (Ningbo Sklon Lab Instrument Co., Ltd. Ningbo, China) at different ratios of oil and alloy (2:1, 5:1, 10:1). To mix the permeable oil and NaK homogeneously, NaK and the permeable oil were mixed using the following parameters: 5-seconds duration, 5-second intervals, 10 repetitions, and 150 W maximum output power. The stabilized uniform microdroplet containing NaK and permeable oil was disinfected using UV light at least 15 minutes before its use in the following tests (Fig 1).

### Animals and tumor model

Ten New Zealand white rabbits weighing between 2.5 and 3.0 kg were used. The protocol was approved by the Committee on the Ethics of Animal Experiments of the Beijing Union Hospital (Permit Number: SYXK20100028), and the study was carried out in strict accordance with the recommendations in the Guide for the Care and Use of Laboratory Animals of the National Institutes of Health. All surgeries were performed under anesthesia using sodium pentobarbital that was administered in the marginal ear vein. The VX2 tumor strain was transplanted into the hind thigh muscles of tumor-carrier rabbits. The tumor was allowed to grow in the thigh muscle for about three weeks before it was transplanted; growth was determined by palpation or CT. The tumor-carrier rabbit was then sacrificed by an overdose of anesthesia, and the VX2 tumor was excised and placed in saline. Necrotic tissues were discarded. The tumor tissue was cut into 1-mm^3^ fragments. Two to three tumor fragments were implanted into the erector spinae of 10 rabbits under CT guidance. The transplanted tumor tissue presented as a local spherical tumor, which was very similar to a solid tumor growth. The transplanted tumor tissue method effectively avoided tumor cell metastasis, which may occur along the needle tract. Erector spinae is relatively large, with a regular shape, which are beneficial for tumor growth and evaluation, and symmetrical to reduce experimental error with high degree of consistency. After 8–10 days, when the implanted tumor mass grew to an average size of 2.0 cm (ranging from 1.5 to 2.5 cm), both plain and contrast enhanced CT scans were performed on the 10 rabbits.

### Visualized ablation of muscle ex vivo using near-infrared and thermal imaging

The infrared Fluke Ti400 Thermal Imager monitored and recorded temperature changes. The imager was positioned above the region of interest (ROI) such that ROI encompassed the entire field of view. The captured thermal image sequences (image frequency, 0.11 Hz), with a spatial resolution of 320 × 240 pixels and a temperature sensitivity of ±0.05°C, were captured for 20 seconds for steady-state measurements and up to 210 seconds during each injection of NaK (total injection volume never exceeded 110 uL and pure NaK value about 10 uL). To obtain recordings of rapid real time temperature changes, thermal image sequences were recorded during the entire ablation process. Thermal image sequences were processed using Fluke SmartView (http://www.fluke.com/) off line.

### NaK-induced curative effects on VX2 tumors *in vivo* using perfusion CT methods

Prior to NaK ablation, CT was used to confirm the tumor location. The surgical site was disinfected with povidone-iodine, after shaving off hair. Perfusion CT was used to compare and evaluate the differences in parameter mappings before and after cancer treatment. A scout scan was performed through the tumor. Axial CT scans without contrast material were used to select the target treatment level of the tumor. Perfusion CT imaging was then performed to acquire the base perfusion details before treatment. The NaK–oil mixture (volume ratio, 1:5, ~10 uL pure NaK) was injected at a depth two-thirds of the target tumor’s diameter using a 22-gauge syringe. The curative effect of the mixture was assessed by a perfusion CT imaging that was performed again after at least 60 minutes, to enable clearing of the contrast material from the preceding acquisition [13].

For the perfusion CT measurements, images of the VX2 tumor in the erector spinae muscles were taken at the selected slice locations using a SOMATOM Perspective CT scanner (Siemens, Erlangen, Germany). Omnipaque 350 (350 mg iodine per mL), a non-ionic contrast medium, was intravenously injected using a Medrad Injector (MEDRAD, INC., Warrendale, PA, USA). After the perfusion imaging levels were identified, imaging was initiated, and 1.5 mL per kg body weight of contrast material was injected at 1.0 mL/sec. Cine CT scans were taken continuously for 90 sec at a speed of 1.0 sec/per rotation (64 sections). All images were acquired using a tube voltage of 120 kVp, a tube current of 80 mA, a field of view of 200 mm, and a section thickness of 5.0 mm [14].

**Fig 2:**
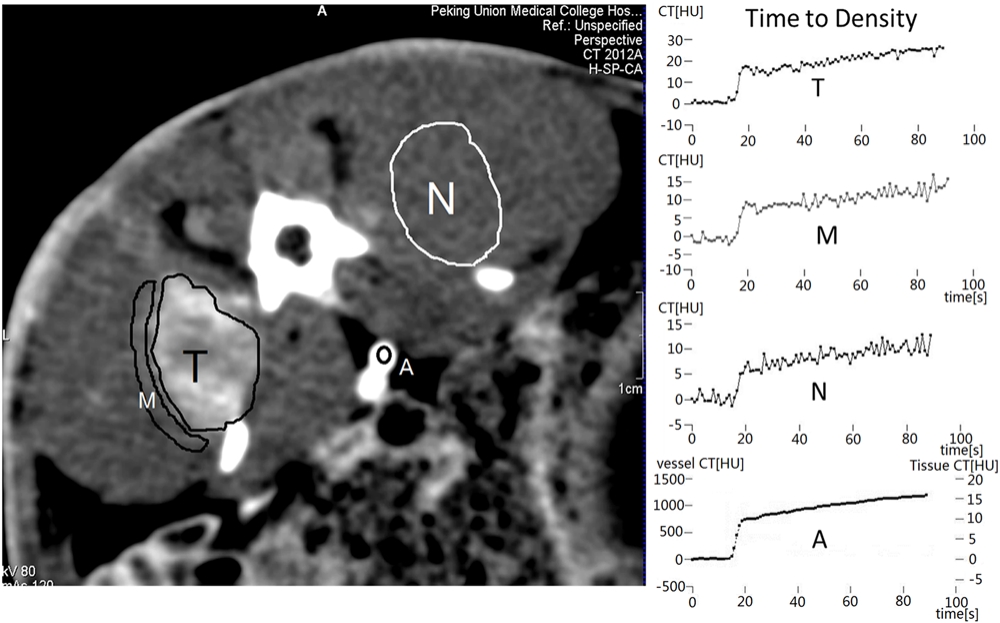
Axial perfusion CT image and time density curve corresponding to the regions of interest: center of the aorta as an arterial input reference (A), around entire tumor (T), around an area of muscle tissue immediately adjacent to the tumor (M), and random location in the normal muscle tissue distant from the tumor (N).

The perfusion images were reconstructed from the raw projection data onto a 512 × 512-pixel matrix before being transferred to a *syngo*.*via* imaging workstation (Siemens Healthcare) for perfusion analysis. Absolute values of blood flow (BF), blood volume (BV), and capillary permeability-surface area (PMB) measurements were determined using CT Body Tumor Perfusion (Siemens Healthcare). At each of the four simultaneously imaged levels, three ROIs were drawn around the entire tumor (T), around an area of normal tissue immediately adjacent to the tumor (M), and in a random location within the normal muscle tissue (N) distant from the tumor as shown in Fig 2. Careful placement of arterial input function region (A) is the basis for accurate determination of CT Perfusion Map Construction, as shown in Fig 2. The BF, BV, and PMB measurements were calculated in each of the three ROIs (T, M, N). All ROIs and perfusion measurements were drawn and obtained by the same investigator. The characteristics of enhancement deduced from the perfusion CT were analyzed offline. All the differences before and after ablation were compared to confirm any recorded changes.

### Trypan blue injection

Trypan blue (TB; Sigma-Aldrich Co., LLC, St. Louis, MO, USA) was dissolved in 10 mM phosphate buffer (pH 7.4) and sterilized by passing through 0.2-μm pore membrane filters. The final dye was 2.0% TB. Animals were injected in the ear vein with approximately 1.0 mL per kg body weight, and then sacrificed by an overdose of anesthetic agent. The animals were visually inspected for dye uptake into the tumor, indicated by blue.

### Histopathology analysis

The tumor and surrounding tissue were excised in order, according to the distance from the central necrosis area. The necrosis area was confirmed by *in vivo* TB injection intravenously into the ear margin. All histopathologic analyses were used to gather evidence of the curative effects of thermal and chemical ablations. The specimens tissues were cut into 5.0 μm sections, processed, and stained with hematoxylin and eosin stains. A senior pathologist performed the histopathology evaluation using microscopy.

### Statistical analysis

Statistical analyses were performed using SPSS for Windows Version 16.0 (SPSS Inc., Chicago, IL, USA). A paired t-test was used to evaluate the statistical significance to compare values before and after treatment during the ablations, and a P-value of <0.05 was considered significant.

## Results

### Measurement of temperature maps and analysis

**Fig 3:**
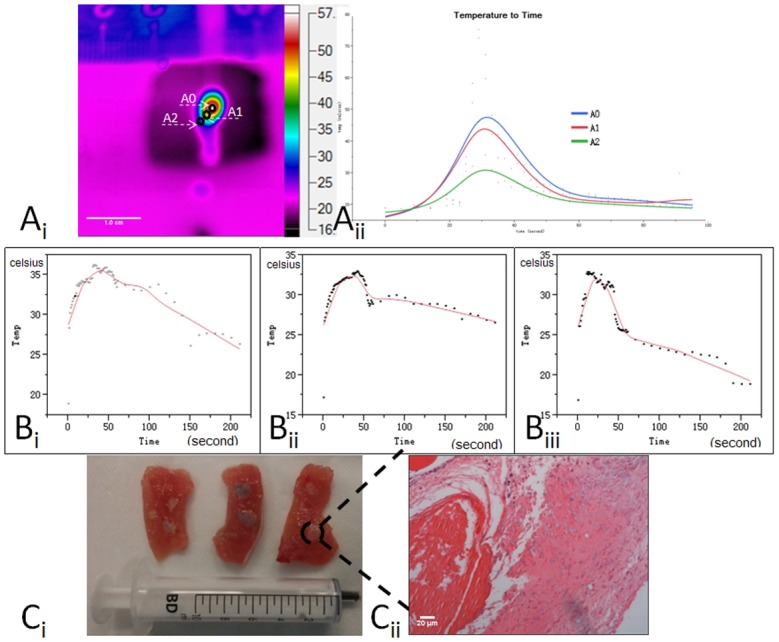
Temperature changes in spatial and temperal relationships during the injection of sodium-potassium alloy-oil mixture. A_i_ and A_ii_) Video stream of the thermal damage as acquired by the infrared Fluke Ti400 Thermal Imager. A0 to A2 indicates the temperature distribution (A_i_) and alteration at different distances from the reaction center (A_ii_). The continuous region of interest selection A0, A1, A2, points in the tissue that the heat readings, and the distance from the center heat source are approximately 0mm (A0), 1mm (A1), 2mm (A2). B_i_, B_ii_, B_iii_) Temperature to time curves were captured from three muscle strips corresponding to different ratios of a mixture of NaK and oil 1:10 (B_i_), 1:5(B_ii_), and 1:2 (B_iii_) for the ablation of muscle with the same amount of NaK. C) From left to right, three muscle strips corresponding to different ratios of a mixture of NaK and oil (1:10, 1:5, and 1:2) for the ablation of muscle. Coming to each muscle stripe, three seperate ablation points with the same volume of the mixture. D) Effects of the NaK–oil mixture on muscle tissue and cells: apparent necrosis of a large number of muscle cells with the disappearance of cell structure and dissolved nuclei.

Temperature changes in spatial and temporal relationships are presented in Fig 3A and 3B. The injection of the NaK–oil mixture resulted in significantly increase in temperature in the ablation areas from 18.93 to 35.90°C (T_max_−T_min_ = 17°C). The instantaneous temperature had been above 75°C at center position; with increasing distance from the center of the reaction, the temperature was lower. The rising slopes and amplitude of the temperature in the ablation area were similar among different ratios of NaK to oil. The most significant rise in temperature was observed within the first 1.0-minute interval, as shown in Fig 3B. When comparing the differences in temperature mapping among the three ratios of NaK to oil, a higher NaK-to-oil ratio (e.g., 1:2) corresponded to a thinner peak of temperature alterations and shorter duration.

The immediate effects of ablation on fresh muscles with the NaK–oil mixture at different ratios had been shown in Fig 3C and 3D. The results suggest that permeable oil significantly controlled the rate of heat released from the reaction between NaK and water in the tissues and induced local necrosis of the cells.

### Establishment of rabbit muscle VX2 tumor model

The VX2 tumor was successfully implanted in the erector spinae in all 10 New Zealand rabbits. The diameter of the tumors was approximately 1.8 cm (range, 1.2–3.1 cm) at 8–10 days after their introduction. The VX2 tumor extruded from the muscle surface and resulted in a hard mass with necrosis at the center of some tumors.

### Perfusion CT examination

**Fig 4:**
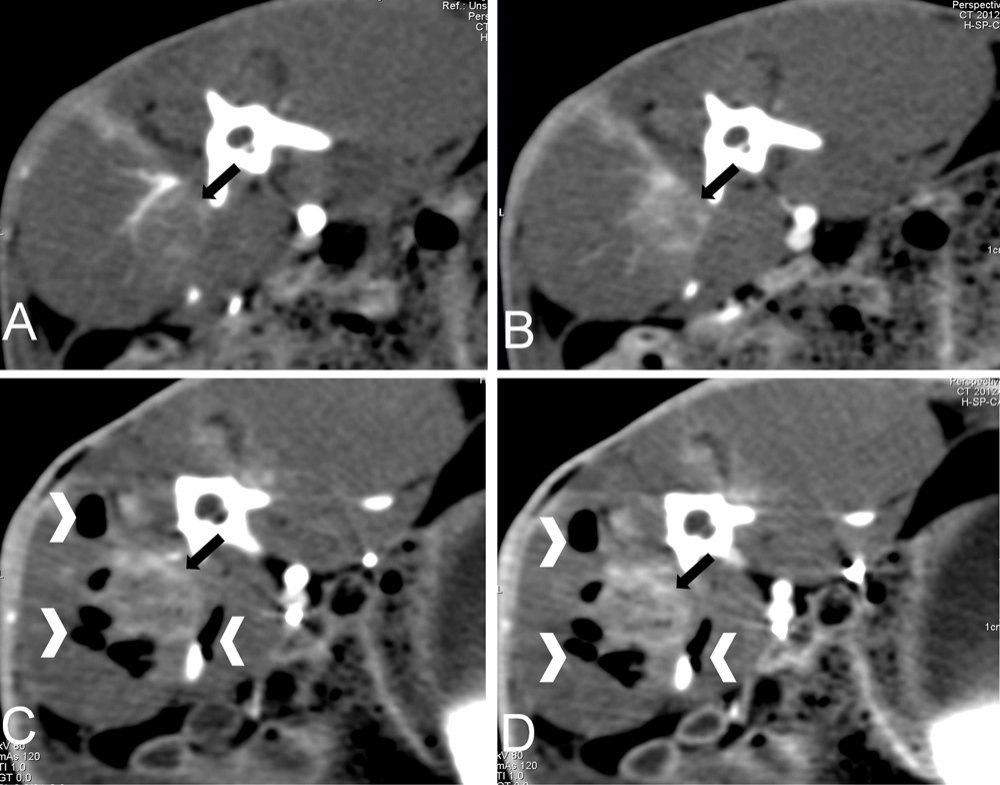
Axial CT images in rabbits show normal muscle tissue and VX2 tumor (black arrow): (A, B) before and (C, D) after NaK–oil mixture (10 μL:50 μL, vol:vol) injection. Baseline images were obtained 15.3 sec (A) and 25.4 sec (B) after the start of contrast material injection, and perfusion images were obtained 15.3 sec (C) and 25.4 sec (D) seconds after start of the NaK–oil mixture injection. In C and D, note the reduced contrast enhancement of the tumor, especially its outer rim. The reaction between NaK and water in the tissue produces a large amount of air (white dovetail) during the exothermic chemical process (C and D).

Both plain and perfusion CT scans were used to differentiate the necrosis area from the viable area to evaluate the curative effects of the NaK-oil ablation treatment for the VX2 tumor *in vivo*. The post-perfusion CT examinations were performed after an interval of >1.0 hour after the first CT. The gas density displayed by CT scan indicated the area where the reaction of NaK with water occurred in the tissues. Comparing perfusion CT before and after treatment, the post-treatment CT showed significantly reduced BF, BV, and permeability of the tumor, which was lowest at the center of the ablated lesion (Fig 4, Table 1).

**Table 1:** Perfusion CT measurements before and after treatment in VX2 tumor (n = 10).

	BF(mL·min^-1^)		BV (mL·min^-1^·100g^-1^)		PMB (mL·min^-1^·100gm)	
Position	Before	Post	Before	Post	Before	Post
N	10.31±0.31	9.05±1.17	1.94±0.49	2.08±0.75	2.77±0.47	2.67±0.95
M	2.06±0.75*	1.23±0.42	3.46±1.45*	1.32±0.64	5.55±1.37*	3.08±0.77
T	54.50±10.79*	23.58±6.95	18.16±4.30*	7.65±2.16	8.02±1.72*	5.21±1.12

* *P* < 0.05

BF = blood flow, BV = blood volume, PMB = permeability surface area N, normal muscle tissue; M, muscle adjacent to tumor; T, tumor

### Histopathologic examination

**Fig 5:**
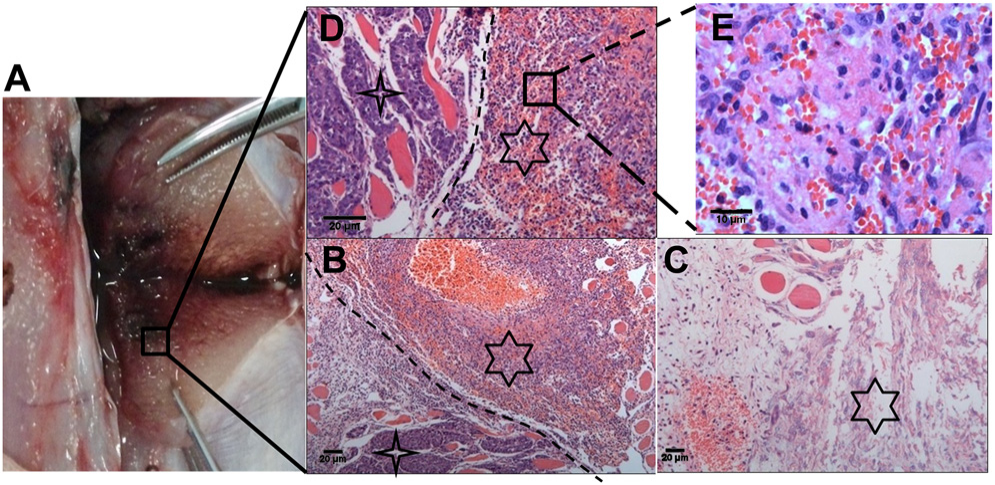
Histopathology analysis of the VX2 tumor. A) Viable cells exclude Trypan blue (TB), while dead cells stain blue from TB uptake. B) Region near the corners (star points) demonstrates areas with integrated tumor structure, while hexagram shows hemorrhagic necrosis areas, X 100. C) Region demonstrates nucleus of tumor cells was completely dissolved and necrotic, X 100. D) Region near corners (star points) demonstrates integrated tumor structure, while hexagram shows hemorrhagic necrotic areas, X 200. E) Regions demonstrate complete karyolysis of tumor cells and a large amount of red blood cell leakage, X 400. TB staining of VX2 tumor after treatment with NaK–oil mixture *in vivo* shows damage to the tissue structure of tumor, which had been further verified by pathology Hematoxylin/eosin staining.

The NaK–oil ablation showed typical signs of coagulative necrosis, including pyknotic nuclei and streamlined cytoplasm (Fig 5). There was vascular thrombosis and hemorrhagic changes in the cells and/or necrosis, as shown in Fig 5B–5E. The VX2 tumor treated with an extremely small amount of NaK–oil mixture shows evidence of cell death and necrosis. These results indicated that permeable oil-controlled release of Na/K could be effective in the treatment of treat tumors.

## Discussion

To our knowledge, this is first report on the use of a liquid alkali alloy sealed with permeable oil in the treatment of tumors *in vivo*. Furthermore, CT and pathological examinations demonstrated the powerful thermochemical ablation effects of the NaK-oil mixture on tumors *in vivo*. Temperature to time curves showed roughly similar changes between different ratios of sodium-potassium (NaK) to oil. There was no significant difference in the peak temperature; rise and decline of the curve became steeper as the ratio of NaK to oil increased, which indicated that permeable oil could effectively control the chemical reaction rate between NaK and water in the tissues, as shown in Fig 3. The findings of the perfusion CT scans correlated with histological results, as shown in Fig 4 and Fig 5, and both methods provided evidence of the effect on the treated tumors. These results suggest that there was an irreversible tissue damage and a clear demarcation between the damaged and undamaged areas (Fig 5B). Histology also demonstrated the dissolution of the nuclei, which identified common markers of thermal damage. The NaK–oil mixture ablations had both thermal and chemical effects during treatment, which generated significant curative effects on the tumor tissue.

Tumor ablation based on exothermic chemical reactions is a new field of treatment with great potential [15,16]. Using the appropriate chemicals as a heat source would be attractive for the possible advantages in safety and effectiveness, and, especially, cost and availability. Further benefits may be derived if the reaction product(s) had local cytotoxic effects without systemic toxicity. Based on these requirements, alkali metals gain special interest as reagents in thermochemical ablation. Alkali metals can be used as heat generating agents in the target tissues, and only an extremely small amount (only about 10 uL pure NaK were used in this study) is necessary to cause a significant increase in temperature or even combustion at the target site, which immediately results in a powerful thermal ablation effect on a tumor. This effect is a result of the strong exothermic reaction between an alkali metal and the intrinsically existing water in the target tissues, as shown in Eq 1.

The reaction between NaK and water can be quantitatively calculated using the following equations:

$$\begin{array}{l} Na+H_{2}O=NaOH+\frac{1}{2}H_{2}↑+140.886KJ/mol\\ K+H_{2}O=KOH+\frac{1}{2}H_{2}↑+140.008KJ/mol \end{array}$$ (1)

Based on an energy conservation relationship, the main factors that quantitatively describe the heat generated from alkali metal thermal ablation could be:

$$\Delta T=\frac{\sum Q}{V_{t}\left(1-\phi \right)\rho_{t}\eta }$$ (2)

*Q* heat of the chemical reaction (W);

∑ total heat release from a subsequent series of reaction;

*ν*_*t*_ volume of target tissue (m3);

*ϕ* volumetric ratio of metal in the total tissues;

*ρ* density of tissue (kg/ m3);

*η* heat release efficiency of exothermic reaction;

Thus, it is evident that the reaction between a pure alkali metal and water in tissues was so intense that an appropriate package is warranted for the NaK mixture to render it safe. Heat-release efficiency during an exothermic reaction *η* might not be a constant. Rather, it is related to the amount of alkali metal used and the interface area between the alkali metal and water in the tissues.

These results encouraged us to explore the potential applications based on the above principles. Compared with a pure alkali metal, NaK remains a liquid at room temperature. NaK can be readily sealed and packaged with other agents, such as oil. It was determined that FMS-121 is stable in heat and to chemical agents, and FMS-121, which is also permeable to water, was used in protein crystallization [12]. As presented in Fig 3, the present study illustrated that the heat produced from NaK and water could be controlled by mixing NaK with a permeable oil. The results of the current experimental design suggested a temperature fluctuation that was influenced by the ratio of the NaK to oil. Using the changes in the ratio of NaK to permeable oil, the alterations in the available water in tissue was adopted as a tool to control heat generation.

However, prior to clinical application, these results have to be confirmed for safety [17]. In this study, a safe administration of the NaK–oil mixture was enabled through a simple injection. The biocompatibility of a fluorosilicone, such as FSM-121, has been proved in other studies, which provided the evidence that silicone oil is still the most biocompatible material [18]. After the NaK–oil mixture was fully emulsified using ultrasound to fully mix and reduce its viscosity, the NaK itself never had contact with the tissue along the insertion path; therefore, it could not release heat until it was injected into the target tissues.

This highly convenient and effective treatment protocol indicates that the thermal ablation method is safe and convenient. Although the clinician needs only to operate with a syringe, as indicated in our study, the structure of the syringe in clinical practice needs to be carefully designed. In addition, a large amount of gas was produced during the treatment, as shown in Fig 2; therefore, in clinical practice, an exhaust pipe should be placed at the reaction site to funnel the released gases into the external environment.

Recently, a comparable study was proposed by Cressman & Jahangir [19], who tested a dual-mode single-agent thermochemical ablation using the simultaneous release of heat energy and acid acetyl from the reactions between acetyl chloride (AcCl) and acetic anhydride. He reported that the reaction of electrophiles in the tissues shows that the new thermochemical ablation technique using a single reagent is potentially useful. In spite of the small volume and relatively low concentration of AcCl, the corresponding coagulation volumes and observed temperature changes were significant. Since they reported the thermochemical ablation in ex vivo liver, the perfusion effects were absent and tissue viability studies were not applicable. A thorough evaluation of the differences between the dual-mode single-agent thermochemical ablation using AcCl and the alkali metal ablation method is not yet available; however, each method has its own merits and warrants further study.

There were several limitations of this study. First, an unrefined, prototype syringe was used to inject the NaK-oil mixture, but this type of end-hole needle is not optimal for this type of therapy. The use of a multiple-side-hole device would most likely improve the results [20]. Second, although ethanol and acetic acid have been used in clinical practices [6,7], the chemical reaction mechanisms involved in treatment are far different from the thermal chemical reactions between alkali metal and water. Pure NaK alloy has induced severely exothermic reaction and combustion phenomena within tissues [9], which is different from that observed in this study. Although gelatin packaged solid alkali metal had been used for the of thermal ablation experiments [10], the reaction between the solid metal and gelatin are significantly different from this study. So far, because of the lack of a well-recognized thermochemical treatment for tumor ablation as a reference standard, this study was designed to compare the conditions before and after treatment with the NaK–oil mixture. Third, only short-term tissue-perfusion data were available because this preliminary research was designed to be a terminal study. The perfusion reduction could have been a result of transient vascular changes, such as a reactive vasospasm, or from permanent tissue destruction from the ablation. Future experiments wherein perfusion CT is repeated several days after the ablation to elucidate the extent of the perfusion changes are required. Fourth, infrared mapping can only record the surface temperature; the temperature changes that occurred in the deep tissues have not been measured. In the future study, we propose to record multi-point temperature response using a local thermocouple probe. Finally, the present study was a proof of the concept study to confirm if tumor ablation can be done using liquid alkali metals. Future work will address some of these issues to elucidate the detailed mechanisms of the action and quantitative assessment of time-effects pattern of tumor tissue ablation.

In conclusion, the controlled heat-producing reaction resulting from the contact of water within the tissues of the mixture of permeable oil with a liquid alkali metal shows promise as a new thermochemical ablation technique. The temperature changes, perfusion maps, and necrosis observed in the *in vivo* tumor model show potential for the use of permeable oil-packaged liquid alkali metals in thermochemical ablation.
